# Changes in DNA methylation from pre- to post-adolescence are associated with pubertal exposures

**DOI:** 10.1186/s13148-019-0780-4

**Published:** 2019-12-02

**Authors:** Luhang Han, Hongmei Zhang, Akhilesh Kaushal, Faisal I. Rezwan, Latha Kadalayil, Wilfried Karmaus, A. John Henderson, Caroline L. Relton, Susan Ring, S. Hasan Arshad, Susan L. Ewart, John W. Holloway

**Affiliations:** 1 Department of Mathematical Sciences, University of Memphis, Memphis, TN 38152 USA; 2 Division of Epidemiology, Biostatistics, and Environmental Health, University of Memphis, Memphis, TN 38152 USA; 3 School of Medicine, Emory University, Atlanta, GA 30322 USA; 4 School of Water, Energy and Environment, Cranfield University, Cranfield, Bedfordshire MK43 0AL UK; 5 Human Development and Health, Faculty of Medicine, University of Southampton, Southampton, SO17 1BJ UK; 6 Population Health Sciences, Bristol Medical School, University of Bristol, Bristol, BS8 1QU UK; 7 MRC Integrative Epidemiology Unit at the University of Bristol, Bristol, BS8 1QU UK; 8 Clinical and Experimental Sciences, Faculty of Medicine, University of Southampton, Southampton, SO17 1BJ UK; 9 David Hide Asthma and Allergy Research Centre, St Mary’s Hospital, Newport, Isle of Wight PO30 5TG UK; 10 College of Veterinary Medicine, Michigan State University, East Lansing, MI 48824 USA

**Keywords:** DNA methylation, Stability, Gender, Epigenetic, Puberty, Adolescence, Whole-genome, IOW, ALSPAC

## Abstract

**Background:**

Adolescence is a period characterized by major biological development, which may be associated with changes in DNA methylation (DNA-M). However, it is unknown to what extent DNA-M varies from pre- to post-adolescence, whether the pattern of changes is different between females and males, and how adolescence-related factors are associated with changes in DNA-M.

**Methods:**

Genome-scale DNA-M at ages 10 and 18 years in whole blood of 325 subjects (*n* = 140 females) in the Isle of Wight (IOW) birth cohort was analyzed using Illumina Infinium arrays (450K and EPIC). Linear mixed models were used to examine DNA-M changes between pre- and post-adolescence and whether the changes were gender-specific. Adolescence-related factors and environmental exposure factors were assessed on their association with DNA-M changes. Replication of findings was attempted in the comparable Avon Longitudinal Study of Parents and Children (ALSPAC) cohort.

**Results:**

In the IOW cohort, after controlling for technical variation and cell compositions at both pre- and post-adolescence, 15,532 cytosine–phosphate–guanine (CpG) sites (of 400,825 CpGs, 3.88%) showed statistically significant DNA-M changes from pre-adolescence to post-adolescence invariant to gender (false discovery rate (FDR) = 0.05). Of these 15,532 CpGs, 10,212 CpGs (66%) were replicated in the ALSPAC cohort. Pathway analysis using Ingenuity Pathway Analysis (IPA) identified significant biological pathways related to growth and development of the reproductive system, emphasizing the importance of this period of transition on epigenetic state of genes. In addition, in IOW, we identified 1179 CpGs with gender-specific DNA-M changes. In the IOW cohort, body mass index (BMI) at age 10 years, age of growth spurt, nonsteroidal drugs use, and current smoking status showed statistically significant associations with DNA-M changes at 15 CpGs on 14 genes such as the *AHRR* gene. For BMI at age 10 years, the association was gender-specific. Findings on current smoking status were replicated in the ALSPAC cohort.

**Conclusion:**

Adolescent transition is associated with changes in DNA-M at more than 15K CpGs. Identified pathways emphasize the importance of this period of transition on epigenetic state of genes relevant to cell growth and immune system development.

## Background

The time period from pre-adolescence to post-adolescence is denoted as the adolescence transition period, during which children experience significant gender-dependent social, environmental, and physiological changes, e.g., transition to independence, initiation of smoking, puberty, rapid growth, and often body mass index (BMI) increase [1–4]. The timing and intensity of these changes have been associated with a range of adult phenotypes. For instance, we and others have found that the timing and intensity of pubertal events are associated with height [3, 5] and lung function [6] in adulthood. In addition to physiological changes, pre- and post-adolescence transition has also been linked to changes in disease status for conditions such as asthma, emphasizing the importance of this critical transition period to life-long health. In particular, a gender reversal of asthma prevalence occurs across adolescence from male predominance of asthma prevalence in pre-adolescence to female predominance in post-adolescence, primarily as a consequence of increased new onset and reduced remission in girls [7–10].

It is known that epigenetic factors regulate cell lineage and tissue-specific gene expression, and one of the most commonly studied epigenetic mechanisms is DNA methylation (DNA-M). DNA-M is a covalent addition of a methyl-group to a methyl group to a cytosine followed by a guanine (cytosine–phosphate–guanine or CpG). Reduction of DNA methylation may facilitate transcription through allowing transcription factors or co-activators to bind to regulatory elements (promoter or enhancer regions) [11–13]. At intragenic regions, DNA-M has inverted U-shape relationship with gene expression levels, whereby the highest levels of intragenic methylation are found in moderately expressed genes [11, 14]. Through epigenetic regulation of gene activity, DNA-M is associated with disease susceptibility directly, or through synergistic effects with single nucleotide polymorphisms [15–17].

Longitudinal studies have established that DNA-M over time is stable at some CpG sites and varies at others (dynamic methylation) [18–21]. In our recent study, we also demonstrated changes in DNA-M between pre- and post-adolescence in genes encoding components of the Th2 immune response pathway and their association with asthma status change [22]. Given the regulatory function of DNA-M and its association with health conditions, assessing changes of DNA-M in a longitudinal setting provides the potential to identify epigenetic biomarkers of disease-susceptibility. A recent study based on 55 children (30 girls) examined changes in DNA-M between ages 8 and 14 years and identified 48 CpGs that DNA-M showed statistically significant changes regardless of gender and 397 gender-specific CpGs [23]. However at age 14, most children are still in the adolescence transition period; hence, the CpGs identified at this age may not be truly representative of pre- and post-adolescence transition phenomena. Changes in DNA methylation in response to environmental exposures that alter in the adolescent period have also been identified, for example, the use of oral contraceptives and age at menarche with DNA-M at age 18 in the *GATA3* gene [1]. Identification of such changes in DNA methylation will lead to increased understanding of the mechanisms by which such modifiable factors may lead to change in phenotype and provide new opportunities for intervention.

In this study, the aim was to identify CpGs at the genome scale where DNA-M significantly changes from pre-adolescence to post-adolescence (denoted as “dynamic CpGs”) and assess their gender-specificity. Association of these dynamic CpGs with adolescence-related factors such as growth spurt and body mass index, as well as exposures such as tobacco smoke and air pollution were assessed. We hypothesized that DNA-M changes from pre- to post-adolescence and adolescence-related factors were associated with these changes.

## Results

Subjects in the Isle of Wight (IOW) birth cohort with DNA-M available at both ages, 10 years (pre-adolescence) and 18 years (post-adolescence), were included in the study (*n* = 325 including 140 female participants). We analyzed in total 400,825 CpG sites common to Illumina 450k and 850k EPIC array platforms.

### Results from the IOW birth cohort

At the genome-scale, medians of DNA-M at all CpGs (in *M* values calculated as base 2 logit transformed beta values) indicated that DNA-M at age 18 tended to be higher than that at age 10 years for both genders, but at age 18 DNA-M of females overall was higher than that of males (Table 1).

**Table 1:** Descriptive statistics of *M* values (IOW, 400825 CpGs)

Gender		Age 10	Age 18
Female (*n* = 140)	Median	0.72	0.85
	95% empirical interval	− 7.18, 4.17	− 7.08, 4.85
Male (*n* = 185)	Median	0.73	0.79
	95% empirical interval	− 7.03, 4.02	− 7.03, 4.44

**Fig. 1:**
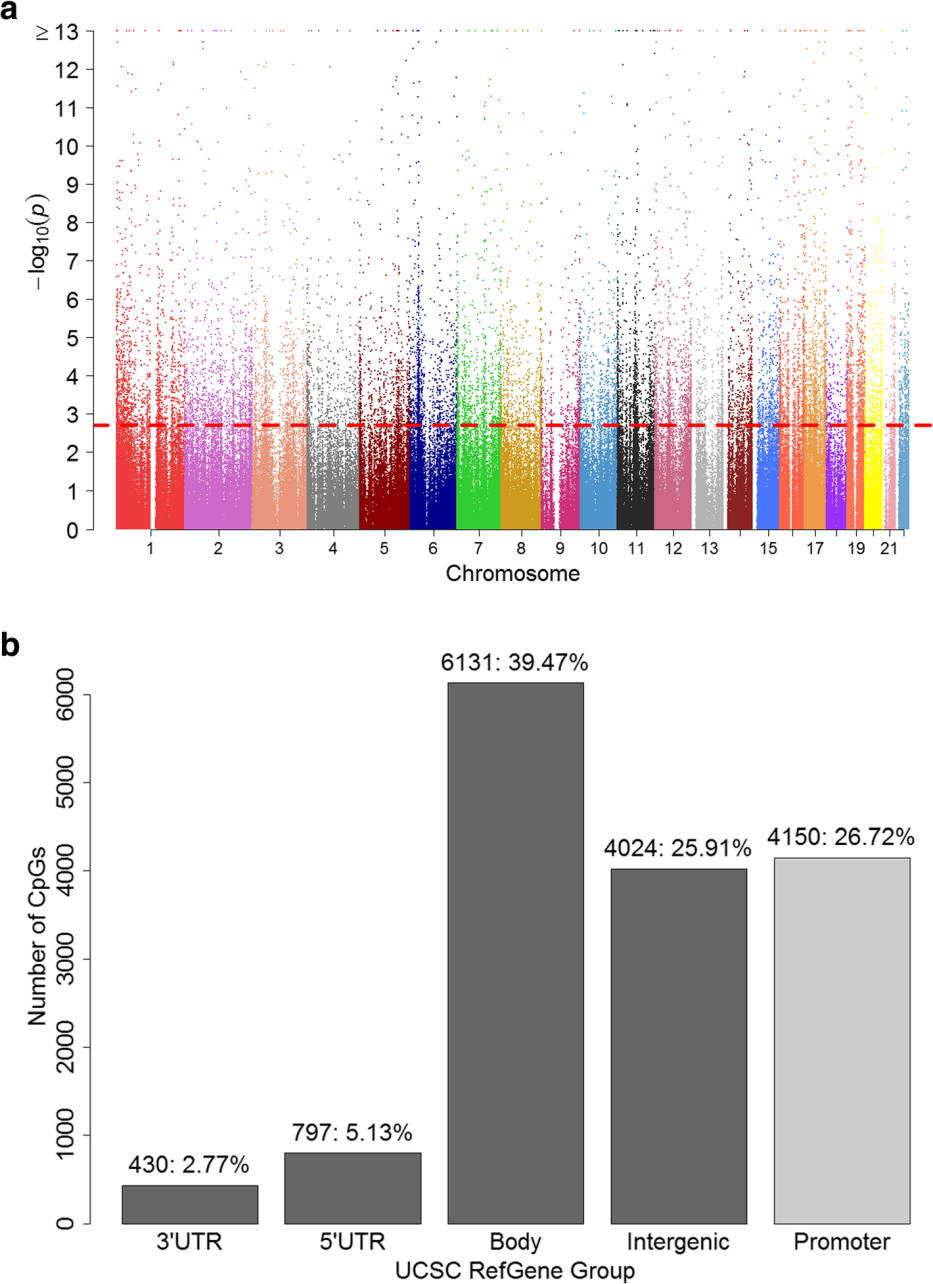
**a** Manhattan plot of Model 1 (examining the overall changes of DNA-M from pre- to post-adolescence. The *x-*axis represents chromosome numbers [1 to 22]. The *y-*axis is −log10 transformed raw *p* values (−log_10_*p*). CpGs with FDR-adjusted *p* value less than 0.05 were located above the dotted line. **b** Locations of the identified CpGs Model 1 (gender invariant DNA-M changes), *n:%*. Body includes the region of body and 1st Exon, and the promoter region includes TSS1500 and TSS200

With respect to individual CpG sites, when focusing on DNA-M changes (from pre- to post-adolescence) without considering gender specificity (Model 1, see Methods section), 15,532 (~ 3.88%) of the 400,825 CpGs showed statistically significant changes (dynamic CpGs). Of these 15,532 CpGs, DNA-M increased from age 10 to age 18 at 8894 (57%). The Manhattan plot of −log10 transformed *p* values of the 400,825 tests are shown in Fig. 1a with the dashed line indicating the cut-off of statistical significance after controlling false discovery rate (FDR) of 0.05. Approximately 1/3 (27%) of the dynamic CpGs were located in gene promoters within 200 bp (TSS200) or 1500 bp (TSS1500) of the transcription start site (Fig. 1b), significantly lower than the proportion of total CpGs (29%) in the analysis (*p* value = 2.0 × 10^−10^).

**Fig. 2:**
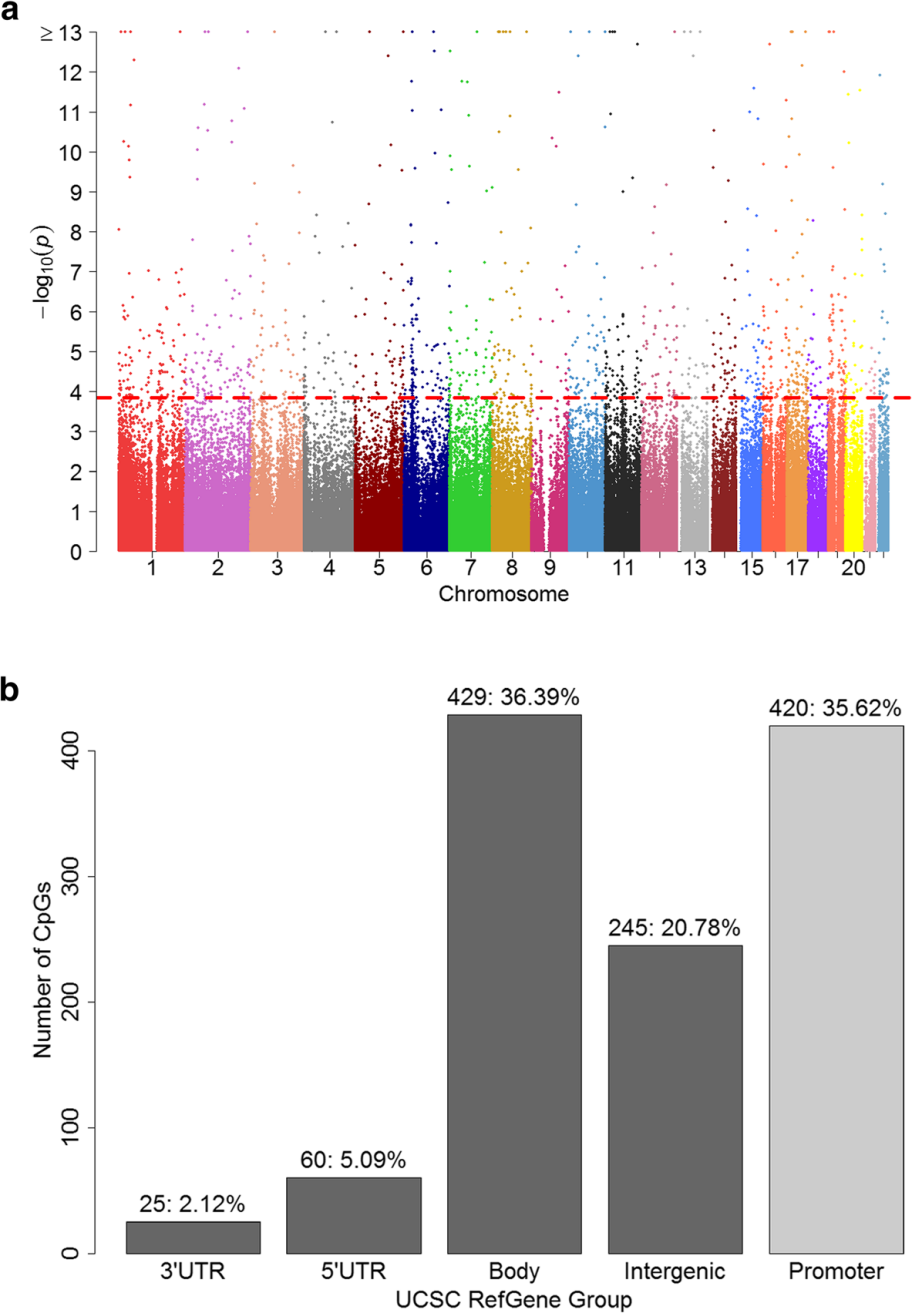
**a** Manhattan plot of Model 2 (examining gender-specific changes of DNA-M from pre- to post-adolescence). The *x-*axis represents chromosome numbers [1 to 22]. The *y-*axis is −log10 transformed raw *p* values (−log_10_*p*). CpGs with FDR-adjusted *p* value less than 0.05 were located above the dotted line. **b** Locations of the identified CpGs Model 2 (gender-specific DNA-M changes), *n:%*. Body includes the region of body and 1^st^ Exon, and the promoter region includes TSS1500 and TSS200

Using linear mixed models with gender and time interaction effects included (Model 2), we identified at 1179 CpGs (FDR = 0.05; Additional file 1), where DNA-M changes across adolescence were gender-specific (Fig. 2a). More than 36% (420 CpGs) of these 1179 gender-specific dynamic CpGs were in promoter regions (Fig. 2b), significantly higher than the percentage (27%) of gender non-specific dynamic CpGs (*p* value = 41.6 × 10^−11^). A small portion of the 1179 CpG sites (83 CpGs; 7%) were among the 15,532 dynamic CpG sites identified without assessing gender specificity, implying that DNA-M at the remaining 1096 CpGs did not show an overall change pre- and post-adolescence (irrespective of gender), but DNA-M changed at least in one gender. Of the 1179 CpGs, for both genders, DNA-M at 265 CpG sites was higher at age 18 years, and lower at age 18 years at 469 CpGs, but the magnitude of change was different between females and males. For the remaining 445 CpG sites (of the 1179), the direction of methylation changes was opposite between females and males.

### Results from the replication cohort, Avon Longitudinal Study of Parents and Children

To assess reproducibility of the findings from the IOW cohort the same models at these CpGs used in the IOW cohort were applied to data from the Avon Longitudinal Study of Parents and Children (ALSPAC) cohort. After controlling for FDR of 0.05, of the 15,532 dynamic CpGs identified in the IOW, 13,739 (~ 88%) were found to be statistically significant in ALSPAC. Among these 13,739 dynamic CpG sites, consistent directions of association as those in the IOW cohort were observed at 10,212 CpG sites (74%). The distributions of the CpGs with respect to direction of change and genomic location were also comparable to those found in the IOW cohort; for instance, 27% (2757 CpGs) of the 10,212 CpGs were in the promoter region of genes, and DNA-M at 61% CpGs (6199 CpGs) was higher at age ~ 15.5 years compared with ~ 7.5 years of age. For the 1179 gender-specific dynamic CpGs identified in the IOW cohort, 37 (~ 3%) of them survived multiple testing in the ALSPAC cohort at FDR = 0.05. For both genders, none of the 37 CpGs showed the same direction of associations as those in the IOW cohort.

### Pathway analysis of replicated dynamic CpG sites

**Fig. 3:**
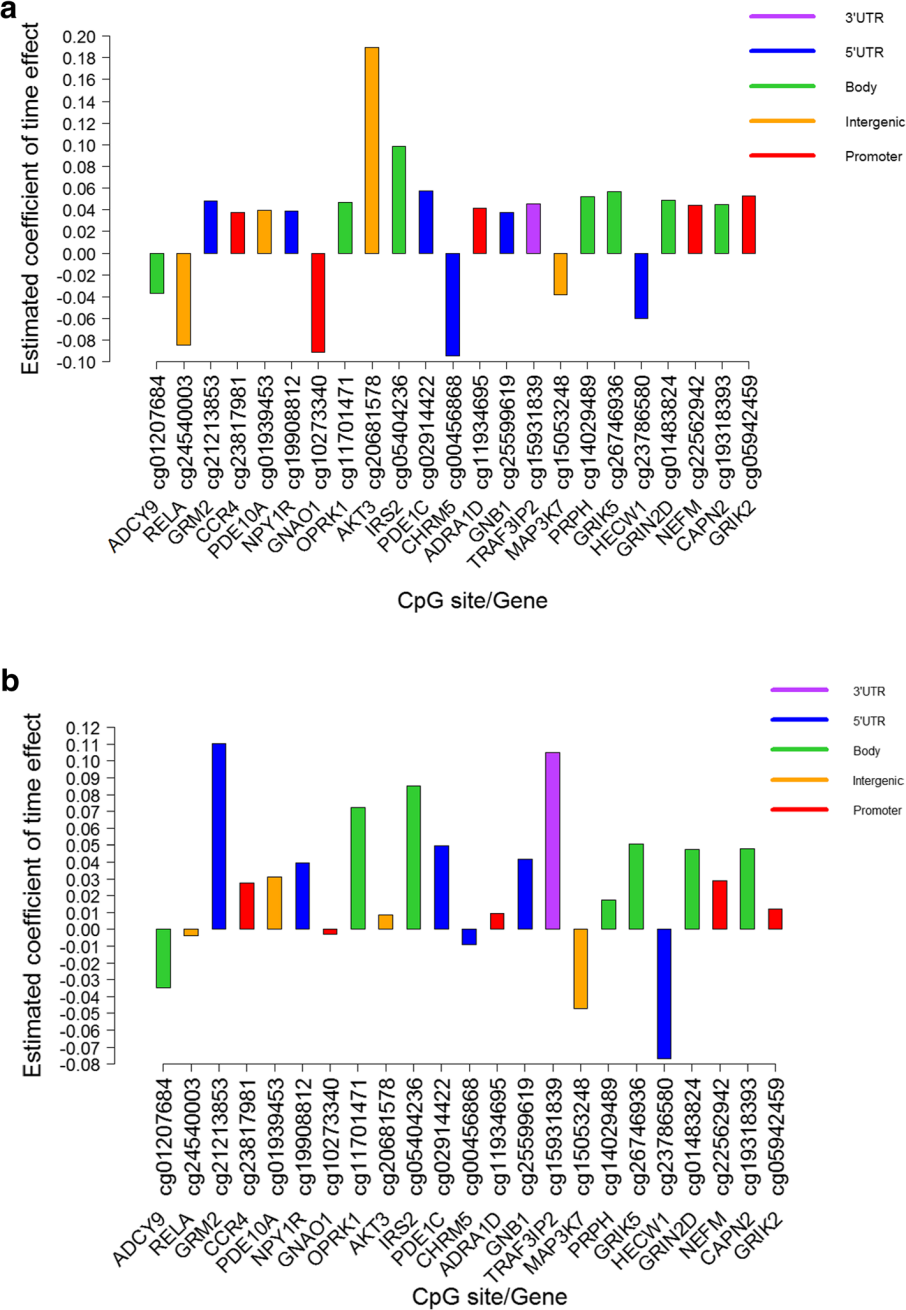
**a** Estimated changes of DNA-M between pre- and post-adolescence on CpGs linked to pathways in Table 2 (IOW). **b** Estimated changes of DNA-M between pre- and post-adolescence on CpGs linked to pathways in Table 2 (ALSPAC)

We applied pathway enrichment analysis to the top 500 dynamic CpGs (of the 10,212 CpGs replicated in ALSPAC) selected by effect size in the IOW cohort. Pathway analyses using Ingenuity Pathway Analysis (IPA) [24] revealed 56 significant canonical pathways (*p* < 0.05) (Additional file 2). Among them the most statistically significant four pathways were Amyotrophic Lateral Sclerosis Signaling, G-Protein Coupled Receptor Signaling, Relaxin Signaling and IL-17A Signaling in Airway Cells (Table 2). In total, 23 identified CpG sites were mapped to the genes in these four pathways. Figures 3 a and b show the changes of DNA-M at these 23 CpGs in both IOW and ALSPAC cohorts, along with the location of the CpGs with respect to genomic location).

**Table 2:** The four most statistically significant canonical pathways from IPA

Pathways	Ratio	*p* value	CpG sites	Genes
Amyotrophic Lateral Sclerosis Signaling	0.081	0.00026	cg01483824	*GRIN2D*
			**cg05404236**	***IRS2***
			cg05942459	*GRIK2*
			cg14029489	*PRPH*
			cg19318393	*CAPN2*
			**cg20681578**	***AKT3***
			cg22562942	*NEFM*
			cg23786580	*HECW1*
			cg26746936	*GRIK5*
G-Protein Coupled Receptor Signaling	0.050	0.0010	cg00456868	*CHRM5*
			**cg01207684**	***ADCY9***
			**cg01939453**	***PDE10A***
			**cg02914422**	***PDE1C***
			**cg05404236**	***IRS2***
			**cg10273340**	***GNAO1***
			cg11701471	*OPRK1*
			cg11934695	*ADRA1D*
			cg19908812	*NPY1R*
			**cg20681578**	***AKT3***
			cg21213853	*GRM2*
			cg23817981	*CCR4*
			**cg24540003**	***RELA***
Relaxin Signaling	0.057	0.0032	**cg01207684**	***ADCY9***
			**cg01939453**	***PDE10A***
			**cg02914422**	***PDE1C***
			**cg05404236**	***IRS2***
			**cg10273340**	***GNAO1***
			**cg20681578**	***AKT3***
			**cg24540003**	***RELA***
			cg25599619	*GNB1*
IL-17A Signaling in Airway Cells	0.077	0.0036	**cg05404236**	***IRS2***
			cg15053248	*MAP3K7*
			cg15931839	*TRAF3IP2*
			**cg20681578**	*AKT3*
			**cg24540003**	***RELA***

[1] Ratio is the number of genes in our list to the number of genes in a pathway [2]. CpGs and genes identified in at least two pathways are in bold font

### Associations of pubertal exposures with replicated dynamic CpG sites

Adolescence is a period accompanied by significant changes such as puberty, rapid growth, and often BMI increases. We postulated that adolescence related factors were associated with changes in DNA-M (Additional file 3). In this analysis, we focused on the 10,212 CpGs replicated in ALSPAC. Among the adolescence factors of interest, age of growth spurt, frequency of taking non-steroidal drug, and current smoking status were shown to be associated with DNA-M changes at 13 CpGs (FDR-adjusted *p* values < 0.05) and these associations did not differ between females and males. At two CpGs, cg08770870 and cg19663246, BMI at age 10 years was associated with DNA-M changes (FDR-adjusted *p* values < 0.05) and the associations were different between genders (Table 3). The influence of BMI at age 10 years on DNA-M at age 18 years (adjusting for the effect of age 10 years’ DNA-M) was different between the two genders; at cg08770870, high BMI was linked to lower DNA-M post-adolescence for girls, but higher post-adolescence DNA-M among males (interaction *p* value = 1.78 × 10^−6^), while at cg19663246, high BMI was associated with high DNA-M post-adolescence for both females and males, but for males, DNA-M was higher (interaction *p* value = 3.86 × 10^−6^). For a subject with later age of growth spurt, post-adolescence DNA-M at cg08134671 tended to be lower (*p* values = 7.15 × 10^−7^). For subjects who used non-steroidal drugs more often, post-adolescence DNA-M at three CpGs (cg16384862, cg18552620, cg12170787) was lower compared with subjects who rarely used such drugs (all raw *p* values < 10^−5^). Similar patterns were observed among subjects who were active smokers; at all 9 identified CpGs, post-adolescence DNA-M was lower if a subject was exposed to tobacco smoke (all *p* values < 10^−4^ with smallest *p* value = 1.0 × 10^−13^, Table 3).

**Table 3:** Analysis of the effect of pubertal exposures on dynamic CpG sites replicated in ALSPAC

Pubertal exposures		CpG sites	Genes	Est. coeff.	*p* value_Raw_	*p* value_Adj._
BMI_10_	BMI_10_ × gender^**$**^	**cg08770870**	***RPH3AL***	**0.060**	**1.78 × 10**^−**6**^	**0.018**
		**cg19663246**	***PDE1C***	**0.038**	**3.86 × 10**^−**6**^	**0.020**
	BMI_10_	cg08770870	*RPH3AL*	− 0.016	0.013	0.457
		cg19663246	*PDE1C*	0.002	0.70	0.945
Pubertal events	Age growth spurt	**cg08134671**	***GNG7***	**− 0.059**	**7.15 × 10**^−**7**^	**0.007**
	Female’s age of menarche	cg06271237	*PLAGL1*	− 0.028	7.91 × 10^−6^	0.081
Other pubertal exposures	Non-steroidal drugs	**cg16384862**	***CWC15;*** ***KDM4D***	**− 0.161**	**1.23 × 10**^−**7**^	**0.001**
		**cg18552620**	***STK17A***	**− 0.206**	**1.66 × 10**^−**7**^	**0.001**
		**cg12170787**	***SBNO2***	**− 0.014**	**7.60 × 10**^−**6**^	**0.026**
		cg00620824	*HLA-C*	− 0.029	3.93 × 10^−5^	0.080
		cg12054453	*TMEM49*	− 0.035	3.93 × 10^−5^	0.080
		cg11001739	*MAD1L1*	0.029	5.27 × 10^−5^	0.090
	Do you currently smoke (yes)	**cg05575921**	***AHRR***	**− 0.742**	**1.0 × 10**^−**13**^	**3.40 × 10**^−**10**^
		**cg14753356**	***IER3***	**− 0.193**	**1.0 × 10**^−**13**^	**3.40 × 10**^−**10**^
		**cg26703534**	***AHRR***	**− 0.178**	**1.0 × 10**^−**13**^	**3.40 × 10**^−**10**^
		**cg24296397**	***BSN***	**0.094**	**7.25 × 10**^−**7**^	**0.002**
		**cg08709672**	***AVPR1B***	**− 0.098**	**1.09 × 10**^−**6**^	**0.002**
		**cg16391678**	***ITGAL***	**− 0.115**	**8.57 × 10**^−**6**^	**0.015**
		**cg20295214**	***AVPR1B***	**− 0.117**	**1.55 × 10**^−**5**^	**0.023**
		**cg21241889**	***TRAF1***	**− 0.070**	**2.36 × 10**^−**5**^	**0.030**
		**cg04885881**	***SRM***	**− 0.092**	**3.99 × 10**^−**5**^	**0.045**
		cg08109568	*FAN1*	− 0.170	7.43 × 10^−5^	0.071
		cg08884752	*SKI*	− 0.078	7.63 × 10^−5^	0.071

[1] ^$^Female is the reference group [2]. Est. coeff.: estimated regression coefficients for each risk factor [3]. *p* value_Raw_: raw *p* values for each test [4]. *p* value_Adj_: FDR-adjusted *p* values, and FDR-adjusted *p* values < 0.05 were in bold font. CpGs with FDR-adjusted *p* values < 0.1 were also included

We further tested in the ALSPAC cohort the CpGs that were shown to be associated with adolescence factors in IOW. In the ALSPAC cohort, variables available for analysis were BMI at age 5 or 7 years, reported age of menarche, and current smoking status. Of the 9 CpGs identified in IOW to have DNA-M changes associated with current smoking status, DNA-M changes at 8 of these CpGs showed the same direction of association in ALSPAC as those in the IOW cohort, of which 5 (cg05575921, cg14753356, cg26703534, cg08709672, and cg16391678) were statistically significant (*p* value < 0.05, Table 4).

**Table 4:** Pubertal exposure analysis in ALSPAC on the factors and CpGs identified in IOW

Pubertal exposures		CpG sites	Genes	Est. coeff.	*p* value_Raw_
BMI_5/7_	BMI_5/7_ × gender^**$**^	cg08770870	*RPH3AL*	− 0.018	0.465
		cg19663246	*PDE1C*	− 0.002	0.932
	BMI_5/7_	cg08770870	*RPH3AL*	0.008	0.535
		cg19663246	*PDE1C*	0.017	0.175
Pubertal events	Female’s age of menarche	cg06271237	*PLAGL1*	0.004	0.619
Other factors	Current/Former smoking status (Yes)	**cg05575921**	***AHRR***	**− 0.199**	**7.43 × 10**^−**8**^
		**cg14753356**	***IER3***	**− 0.099**	**0.010**
		**cg26703534**	***AHRR***	**− 0.071**	**0.002**
		cg24296397	*BSN*	− 0.017	0.403
		**cg08709672**	***AVPR1B***	**− 0.035**	**0.031**
		**cg16391678**	***ITGAL***	**− 0.092**	**0.003**
		cg20295214	*AVPR1B*	− 0.004	0.896
		cg21241889	*TRAF1*	− 0.029	0.137
		cg04885881	*SRM*	− 0.033	0.236
		cg08109568	*FAN1*	− 0.005	0.868
		cg08884752	*SKI*	− 0.048	0.131

[1] ^$^Female is the reference group [2]. Est. coeff.: estimated regression coefficients for each risk factor [3]. *p* value_Raw_: raw *p* values for each test, and *p* values < 0.05 are in bold font

For the 21 CpG sites showing associations with pubertal exposures in the IOW (Table 3), we further examined their biological evidence using expression data via RNA-seq. Of the 21 CpGs, 18 were available for the analysis. Genes within 500 kbp upstream and downstream of the CpGs were considered neighboring genes. In total 16 genes were identified and included in the analyses to examine the association of DNA-M with expressions of their neighboring genes (Table 5). At 5 CpGs, DNA-M showed statistically significant associations, of which two CpGs (cg05575921 and cg26703534) are on the *AHRR* gene and one CpG (cg21241889) is on gene *TRAF1*. At all these five CpGs, higher DNA-M was associated with lower expression of genes (negative associations). At the other 13 CpGs, the associations were statistically insignificant and at 5 CpGs, tendency in negative associations was also observed. All these 5 CpGs are located in the Body region of genes.

**Table 5:** Association of DNA-M at each of the 18 CpGs with expression of their mapped genes

CpG	Gene	Region	Estimate	SE	*p* value
cg21241889	***TRAF1***	**Promoter**	**− 386.899**	**176.498**	**0.030**
cg00620824	*HLA-C*	Promoter	3723.357	7100.079	0.600
cg05575921	***AHRR***	**Body**	**− 2.583**	**0.772**	**0.001**
cg11001739	***MAD1L1***	**Body**	**− 4.885**	**1.634**	**0.003**
cg26703534	***AHRR***	**Body**	**− 4.989**	**2.224**	**0.027**
cg08770870	*RPH3AL*	Body	2.836	1.584	0.076
cg12170787	*SBNO2*	Body	121.684	89.554	0.176
cg08884752	*SKI*	Body	− 20.270	15.247	0.186
cg08709672	*AVPR1B*	Body	− 41.434	44.148	0.350
cg24296397	*BSN*	Body	− 42.795	56.336	0.449
cg06271237	*PLAGL1*	Body	− 6.530	8.863	0.463
cg16391678	*ITGAL*	Body	− 66.564	91.619	0.469
cg20295214	*AVPR1B*	Body	10.419	27.976	0.710
cg08134671	*GNG7*	5'UTR	2.924	3.432	0.396
cg14753356	***IER3***	**Intergenic**	**− 8651.300**	**2796.589**	**0.002**
cg04885881	*SRM*	Intergenic	67.502	136.346	0.621
cg08109568	*FAN1*	Intergenic	42.010	313.325	0.894
cg19663246	*PDE1C*	Intergenic	0.000	0.000	NaN

[1] DNA-M and expressions via RNA-seq measured at age 26 years [2]. DNA-M at these 18 CpGs are associated with pubertal exposures. [3] CpG sites showing statistically significant results are in bold font

## Discussion

In an effort to gain a better understanding of the dynamics of epigenetic change during the critically important adolescent period, this study focused on the changes in DNA-M between pre- and post-adolescence, and further assessed potential factors that might be associated with such changes. In the IOW cohort, we identified more than 15K CpGs where DNA-M changes from pre- to post-adolescence regardless of gender and at 1179 CpGs the changes were different between genders. Of the 1179 CpGs, a large portion (~ 36%) are located in the promoter region of genes. Most (10,212 CpGs, ~ 66%) of the detected 15,532 CpGs were replicated in the ALSPAC cohort. The findings are also consistent with results in a recent study which focused on DNA-M changes between ages 8 and 14 years [23]. Thompson et al. [23] identified 48 differentially methylated CpGs based on genome-scale DNA-M data in 55 children (*n* = 30 girls), and 43 (90%) of these 48 CpGs were among the 10,212 CpGs replicated between IOW and ALSPAC in this study.

In our data, DNA-M in females overall was higher than that in males at age 18 years in the IOW cohort. On the other hand, higher DNA-M in whole blood in males was noted in another study and it was suggested that the higher DNA-M could be due to the additional X-chromosome in females [25]. This contrast was interesting, since in our study, CpGs on sex chromosomes were excluded to avoid such biasness. The observed difference in DNA-M between gender in the IOW may have been due to a different underlying mechanism. Other studies also suggested significant autosomal differences in DNA methylation at specific CpG series between males and females [26] and the higher level of methylation observed in females undoubtedly reflects this. In addition, higher DNA-M in female compared with boys was negligible at age 10 years, compared with age 18 years. We postulate that at this earlier age, adolescence transition just embarked and gender specificity in DNA-M have not reflected in DNA-M. Sex differences in genome-scale DNA methylation pattern have been observed in various tissues in addition to whole blood [27, 28]. In-depth investigations on higher DNA-M in females on autosome chromosomes after adolescence will be helpful.

The findings from pathway analyses based on the top 500 of the 10,212 CpGs emphasized the importance and uniqueness of the adolescence transition period. The G-protein coupled receptor signaling mediates transmembrane signaling involved in diverse physiological functions including hormone release and actions, and cell growth and differentiation. It also regulates immune system activity and inflammation by chemokine receptors binding ligands, which mediate intercellular communication between cells of the immune system. The relaxin signaling pathway is closely related to the development of the reproductive system. Relaxin produced through this pathway is a polypeptide hormone best known for its connective tissue remodeling, which affects the female reproductive system. It promotes the growth of the cervix, uterus, mammary gland, and nipples [29]. In addition, relaxin may have impact on enhancing sperm motility [30]. It also has a role in regulating pituitary hormone release [31], lung and skin remodeling [32] and can induce inhibition of histamine release [33]. IL-17A signaling in airway cells was also identified. IL-17A is linked to the pathogenesis of several inflammatory and autoimmune diseases [34] including respiratory disorders such asthma where it activates MAPK and JAK/STAT signaling in airway smooth cells [35], leading to eosinophil recruitment and promotion of airway inflammation. Of note, in the adolescence period asthma sometimes remits and the higher prevalence childhood asthma in males gives way to the higher female incidence in adulthood [7–10].

Among all the factors associated with changes of DNA-M across adolescence (invariant to gender), non-steroid drug use and smoke exposure affected the greatest proportion of CpGs in the IOW cohort, and findings on more than half of the CpGs associated with active smoking were replicated in the ALSPAC cohort. Although the identified CpGs linked to the use of non-steroid drugs had not been reported in previous studies, and certainly deserve further investigation, most genes including *HLA-C*, *KDM4D*, *SBNO2*, and *TMEM49* to which these CpGs mapped have shown to be associated with inflammation or been treated as inflammation markers [36–38], implying the potential impact of non-steroid drugs on epigenetic mechanisms underlying hemopoietic differentiation. The CpG site cg05575921 in the *AHRR* gene has shown to be associated with active smoke exposure in various studies [39]. The identified association of DNA-M of cg05575921 with expression of *AHRR* further supports the regulatory functionality of epigenetics. Effects of smoking on DNA-M differentiation at the four CpGs corresponding to *AVPR1B*, *ITGAL*, and *SRM* genes also have been shown in multiple cross-sectional studies [40–42]. For instance, at both cg20295214 (*AVPR1B*) [40, 42] and cg04885881 (*SRM*) [42], earlier studies demonstrated lower DNA-M among smokers. Our findings further indicated a longitudinal impact of active smoking on DNA-M reduction at these CpGs. In addition, although the association of DNA-M at cg21241889 with smoking exposure was not statistically significant in the ALSPAC cohort, the direction of association was consistent with that in the IOW. Being located in the promoter region of gene *TRAF1* and accompanied by the connection of smoking exposure with expression of *TRAF1* [43], the statistically significant association of DNA-M at cg21241889 with expression of *TRAF1* revealed a strong potential of this CpG’s functionally regulatory role.

In addition, the impact of some factors on DNA-M changes was different between females and males in the IOW, e.g., pre-adolescence BMI, although these findings were not replicated in the ALSPAC cohort. In recent cross-sectional studies, it has been shown that DNA-M is associated with BMI [44, 45]. Findings from our study indicated that BMI at pre-adolescence may have the potential to predict DNA-M changes in adolescence at some CpGs, e.g., cg08770870 (*RPH3AL*) and cg19663246 (*PDE1C*). Given the associations between BMI, atopy, and asthma [46–48], and a gender-specific effect of pre-adolescence BMI on DNA-M changes suggested by our study, further investigation into the role of these CpGs in each gender on the development of atopy and asthma may improve our understanding on the epigenetic mechanisms of these health conditions. In the IOW, we showed that DNA-M changes at cg08134671 were associated with the age of growth spurt. This CpG was mapped to the *GNG7* gene, a gene critical to the stabilization or formation of a G protein heterotrimer [49]. The protein was likely to contribute to allergic asthma alterations [50]. Although it has been shown that asthma affects growth spurt [51, 52], to our knowledge, no studies have reported whether and how the age of growth spurt influences the status of asthma. These pubertal exposure-associated CpGs sites have a potential to serve as informative epigenetic markers in future studies on asthma and its related health conditions.

The study has some limitations. First, although we had a great consistency in gender-unspecific dynamic CpGs between the IOW and ALSPAC cohorts, the findings on gender-specific dynamic CpGs were not replicated in the ALSPAC cohort. Specifically, in the ALSPAC cohort, 36 of the 1179 gender-specific dynamic CpG sites identified in IOW also showed gender-specific changes, but the direction of gender-specificity was different from that in the IOW. Such inconsistencies might be due to the differences in the ages when DNA-M was assessed between the cohorts: 10 and 18 years in the IOW and 7 and 15 years in ALSPAC. Since children at ages 14 or 15 years are still in the adolescence transition period and some children may have just started puberty, we postulate that at this age gender specificity may have not been reflected in DNA-M. A further assessment on gender-specificity in large cohorts with ages comparable to IOW is warranted in future studies.

In addition, we focused on personal smoking and did not evaluate the effects of second-hand smoking due to the high correlation between these two exposures. In future studies, joint effects between these two levels of exposures may want to be considered for their impact on DNA-M changes. Finally, tissue specificity in DNA-M has been discussed in animal and human studies [53–55]; for instance, larger variations in DNA-M were observed in saliva compared with DNA-M in blood [55]. The tissue specificity in DNA-M is likely to lead to tissue specificity in DNA-M changes over time. The present study only focused on DNA-M changes in whole blood from pre- to post-adolescence. Any generalizability of the findings should be used with caution. Nevertheless, we hope the findings from our epigenetic study would benefit future investigations in the area of adolescence development and health conditions such as childhood asthma where a gender reversal of asthma prevalence was observed. One example of such type of investigations is to test the impact of the identified pubertal exposure-associated CpGs on the risk of asthma transition from pre- to post-adolescence.

## Conclusions

DNA-M at more than 16K CpG sites is likely to change in the period of adolescence transition in one or both genders. Pathways inferred based on the identified CpGs emphasized the importance of this period of transition. Certain factors, including pre-adolescence BMI, age of growth spurt, non-steroid drug use, and current smoke, are potentially associated with DNA-M changes in adolescence.

## Methods

### The Isle of Wight birth cohort

The Isle of Wight birth cohort was established to study the natural history of asthma and allergies and identify genetic and environmental risk factors, and composed of children born on the IOW, UK, between January 1, 1989, and February 28, 1990. The island is close to the British mainland, is semi-rural, and with no heavy industry. The population is largely of Caucasians (~ 99%). Informed consent was obtained from 1456 out of 1536 (~ 95%) newborns. The 1456 newborns were followed up at ages 1 (*n* = 1167; 80.2%), 2 (*n* = 1174; 80.6%), 4 (*n* = 1218; 83.7%), 10 (*n* = 1373; 94.3%), and 18 (*n* = 1313; 90.2%) years. Parents of each child completed detailed questionnaires regarding asthma and allergy prevalence at every follow-up. Blood samples were collected at ages 10 and 18 years from most participants. Details of the IOW cohort have been described elsewhere [56]. In this study, we focused on the age 10 and 18 year follow-ups.

### DNA methylation

DNA was extracted from whole blood samples using a standard salting out procedure [57]. Genome-scale DNA-M was assessed using the Illumina Infinium HumanMethylation450 BeadChip and MethylationEPIC Beadchip (Illumina, Inc., San Diego, CA, USA), which interrogate > 484,000 and > 850,000 CpG sites, respectively, associated with over 24,000 genes. The bisulfite conversion efficiency on all 10 (*n* = 453) and 18 years (*n* = 520) was estimated for array data from each of the samples. MethyLumiSets were generated by reading raw idat files using either the methylumIDAT function [58] of methyLumi package [59] (450k Infinium Human Methylation BeadChip array data) or using the readEPIC function of wateRmelon [60] (Infinium Methylation EPIC array data). The bscon function of wateRmelon was used for estimating the bisulfite conversion efficiency. The bisulfite conversion efficiency was found to be high with a median of 94.1% (IQR 92.8–95.0) (mean (SD), 93.6% (2.9)). These efficiency rates are comparable with the rates estimated for the EZ-96 DNA Methylation kit used by Illumina platforms [61]. Data pre-processing was undertaken using the CPACOR pipeline [62]. Briefly, intensity values from raw IDAT files were background corrected and assessed for quality. Probes not reaching a detection *p* value of 10^−16^ in at least 95% of samples were excluded. The data were quantile normalized using the R package, *minfi* [63]. Autosomal probes were then extracted and converted to beta values. The beta values represent the ratio of the methylated (M) probe intensities of the sum of methylated (M) and unmethylated (U) probe intensities $\Big( Beta=\frac{M}{U+M+C}$ with constant C = 100 introduced for the situation of too small M+U). Next, principal components (PCs) inferred based on control probes were used to represent latent chip-to-chip and technical variation. Since DNA-M data were from two different platforms, we determined the PCs based on DNA-M at shared control probes. In total, 195 control probes were shared between the two arrays, and used to calculate the control probe PCs with the top 15 to represent latent batch factors [64]. CpG sites common between Illumina 450k platform and EPIC platform were included in the study. To reduce the potential influence of probe SNPs, CpG sites were further excluded if the minor allele frequency of the probe SNP in the Caucasian population at that site is > 7% (i.e., ~ ≥ 10 out of 1456 subjects expected to have the minor allele in the cohort) and the probe SNP was within 10 base pairs of the targeted CpG site.

Beta values close to 0 or 1 tend to suffer from severe heteroscedasticity, and it has been demonstrated that base-2 logit transformed beta values (denoted as *M* values) perform better in differential analysis of methylation levels [65]. In this study, *M* values were used to represent methylation levels in the analysis.

The association of DNA-M assessed from whole blood and pubertal exposures could be confounded by cellular heterogeneity [66, 67]. Hence, there was a need to adjust cell type proportions in whole blood. The method proposed by Jaffe and Irizarry [68] that modified from Houseman et al.’s [69] was recommended [70] to estimate cell proportions and implemented in Bioconductor [71] minfi package.

### Statistical methods

To identify dynamic CpG sites such that DNA-M changes between two-time points (pre- and post-adolescence), linear mixed models with repeated measures were implemented. *M* values of DNA-M at both ages were treated as the response variable; gender (with girls as the reference group) and time were included as predictors (Model 1). Adjusting factors included indicators to different platforms (450K and EPIC), cell type proportions and principal components representing latent factors due to batch effects and technical variation at each time point. To assess gender specificity in DNA-M changes, we extended Model 1 with gender × time interaction included (Model 2). In Model 1, CpG sites with statistically significant time effects were treated as being dynamic CpG sites. In Model 2, sites were selected if statistically significant interaction effects were identified. In both models, multiple-testing was corrected by controlling FDR of 0.05 [72]. The analyses were performed in SAS version 9.4 [73] .For the linear mixed models, SAS seems to have difficulty in handling a large number of missing values. CpG sites were not analyzed if they had > 20% missing values.

To examine the association of a phenotypic factor with changes of DNA-M at each identified dynamic CpG site, linear regressions were applied with DNA-M at age 18 years as the dependent variable and a phenotypic factor and DNA-M at age 10 years as the independent variables. The factors (Additional file 3) included BMI at age 10 years, height at age 10, age of growth spurt, age of pubertal events, frequency of nonsteroidal drugs use, current smoke and ever smoked status, pet exposure at age 10 years, pollution, and status of living on farm. For females, pubertal events included body hair growth, skin change, menarche, and breast growth; for males, body hair growth, skin change, facial hair. The goal was to examine the effects of these factors on DNA-M at age 18 years adjusting for DNA-M at age 10 years, i.e., their effects on the change of DNA-M. In the analyses, DNA-M was first corrected for the effects of batch, cell types, and principal components, and then included in the linear regressions. Gender was included in the model as an adjusting factor. To evaluate gender-specificity with respect to the association of a phenotypic factor with DNA-M changes, we further included a gender and factor interaction effect into the model. Results were regarded as statistically significant with *p* value < 0.05 after adjusting for multiple testing by controlling FDR of 0.05 across all testing CpGs for each phenotypic factor. The analyses were performed in SAS version 9.4 [73].

### Replication in ALSPAC

The findings in the IOW were further tested in the ALSPAC longitudinal birth cohort. All pregnant women (expected date of delivery between April 1, 1991, and December 31, 1992) resident in a defined geographical area in the South West of England were eligible. In total, 14,541 pregnant women were recruited, for obstetric data abstractions and earlier questionnaires. Out of the 14,541 women, 13,761 women were eligible, and further 10,321 out of these eligible women had DNA sampled. Details of ALSPAC have been described elsewhere [74–76]. Please note that the study website contains details of all the data that is available through a fully searchable data dictionary and variable search tool (http://www.bristol.ac.uk/alspac/researchers/our-data/)

Genome-scale DNA-M in the ALSPAC cohort was assessed using the Illumina 450k platform. Based on the availability of DNA-M at two-time points (average ages of 7.5 and 15.5 years), data from 478 females and 461 males were analyzed in our study including some subjects from the ARIES study [77]. The pre-processing of DNA-M included removing batch effects and technical variation, details of which can be found elsewhere [77]. CpGs with detection *p* value ≥ 0.01, and samples were flagged that contained sex-mismatch based on X-chromosome methylation were excluded from the analyses. Phenotypic factors examined include pre-adolescence BMI, age of menarche, and active smoking status.

### Pathway analysis

The genes corresponding to CpGs were identified using the Illumina array manifest gene annotations and SNIPPER (https://csg.sph.umich.edu/boehnke/snipper/) version 1.2. The Ingenuity Pathway Analysis system–IPA® was used to identify global canonical pathways (QIAGEN Inc., https://www.qiagenbioinformatics.com/products/ingenuity-pathway-analysis) [24].

### Assessment on biological evidence using RNA-seq data

To examine the biological evidence of the CpGs associated with phenotypic factors, we utilized DNA-M and expressions via RNA-seq measured at age 26 years of *n* = 139 subjects in the IOW cohort.

DNA-M at age 26 years was analyzed using Illumina Infinium Methylation EPIC Beadchip and pre-processed in the same way as the DNA-M at ages 10 and 18 years. Expression at age 26 years was measured using paired-end (2 × 75 bp) RNA sequencing using the Illumina TruSeq Stranded mRNA Library Preparation Kit with IDT for Illumina Unique Dual Index (UDI) barcode primers following manufacturer’s recommendations. All samples were sequenced second time using exactly the same protocol and for each sample the output from both runs were combined. FASTQC [78] were run to assess the quality of the FASTQ files. Reads were mapped against Human Genome (GRch37 version 75) using HISAT2 (v2.1.0) aligner [79]. The alignment files, produced in the Sequence Alignment Map (SAM) format, were converted into the Binary Alignment Map (BAM) format using SAMtools (v1.3.1) [80]. HTseq (v0.11.1) [81] was used to count the number of reads mapped to each gene in the same reference genome used for alignment. Normalized read count FPKM (Fragments Per Kilobase of transcript per Million mapped reads) were calculated using countToFPKM package (https://github.com/AAlhendi1707/countToFPKM).

Linear regressions were then applied to test the associations of DNA-M in *M* values with the expressions of their corresponding genes to assess their biological evidence. Associations with *p* value < 0.05 were deemed as being statistically significant.

## Supplementary information

**Additional file 1.** List of 1,179 CpGs, DNA-M changes across adolescence were gender-specific (FDR=0.05).

**Additional file 2. **List of 56 significant canonical pathways (*p*< 0.05).

**Additional file 3..** Description of pubertal exposures.

## Data Availability

The datasets used and/or analyzed during the current study are available from the corresponding author on reasonable request*.*

## Abbreviations

ALSPAC: Avon Longitudinal Study of Parents and Children
BMI: Body mass index
CpG: Cytosine–phosphate–guanine
CpGs: Multiple CpG
DNA-M: DNA methylation
FDR: False discovery rate
IOW: Isle of Wight
IPA: Ingenuity Pathway Analysis
PCs: Principal components
